# Biomarkers of Cholestasis and Liver Injury in the Early Phase of Acute Respiratory Distress Syndrome and Their Pathophysiological Value

**DOI:** 10.3390/diagnostics11122356

**Published:** 2021-12-14

**Authors:** Lars-Olav Harnisch, Sophie Baumann, Diana Mihaylov, Michael Kiehntopf, Michael Bauer, Onnen Moerer, Michael Quintel

**Affiliations:** 1 Department of Anesthesiology, University of Goettingen Medical Center, Robert-Koch-Str. 40, 37075 Goettingen, Germany; sophie.baumann@med.uni-goettingen.de (S.B.); omoerer@med.uni-goettingen.de (O.M.); mquintel@gwdg.de (M.Q.); 2 Institute of Clinical Chemistry and Laboratory Medicine of the University Hospital Jena, Am Klinikum 1, 07747 Jena, Germany; diana.mihaylov@med.uni-jena.de (D.M.); michael.kiehntopf@med.uni-jena.de (M.K.); 3 Department of Anesthesiology, University Hospital Jena, Bachstr. 18, 07743 Jena, Germany; michael.bauer@med.uni-jena.de

**Keywords:** ARDS, cholestasis, liver injury, GGT, bile acids

## Abstract

Background: Impaired liver function and cholestasis are frequent findings in critically ill patients and are associated with poor outcomes. We tested the hypothesis that hypoxic liver injury and hypoxic cholangiocyte injury are detectable very early in patients with ARDS, may depend on the severity of hypoxemia, and may be aggravated by the use of rescue therapies (high PEEP level and prone positioning) but could be attenuated by extracorporeal membrane oxygenation (ECMO). Methods: In 70 patients with ARDS, aspartate-aminotransferase (AST), alanin-aminotransferase (ALT) and gamma glutamyltransferase (GGT) were measured on the day of the diagnosis of ARDS and three more consecutive days (day 3, day 5, day 10), total bile acids were measured on day 0, 3, and 5. Results: AST levels increased on day 0 and remained constant until day 5, then dropped to normal on day 10 (day 0: 66.5 U/l; day 3: 60.5 U/l; day 5: 63.5 U/l, day 10: 32.1 U/l), ALT levels showed the exact opposite kinetic. GGT was already elevated on day 0 (91.5 U/l) and increased further throughout (day 3: 163.5 U/l, day 5: 213 U/l, day 10: 307 U/l), total bile acids levels increased significantly from day 0 to day 3 (*p* = 0.019) and day 0 to day 5 (*p* < 0.001), but not between day 3 and day 5 (*p* = 0.217). Total bile acids levels were significantly correlated to GGT on day 0 (*p* < 0.001), day 3 (*p* = 0.02), and in a trend on day 5 (*p* = 0.055). PEEP levels were significantly correlated with plasma levels of AST (day 3), ALT (day 5) and GGT (day 10). Biomarker levels were not associated with the use of ECMO, prone position, the cause of ARDS, and paO_2_. Conclusions: We found no evidence of hypoxic liver injury or hypoxic damage to cholangiocytes being caused by the severity of hypoxemia in ARDS patients during the very early phase of the disease. Additionally, mean PEEP level, prone positioning, and ECMO treatment did not have an impact in this regard. Nevertheless, GGT levels were elevated from day zero and rising, this increase was not related to paO_2_, prone position, ECMO treatment, or mean PEEP, but correlated to total bile acid levels.

## 1. Introduction

Impaired liver function and cholestasis are frequent findings in critically ill patients [1] and are associated with poor outcomes [1,2,3,4,5,6,7,8]. Hypoxia of hepatocytes has been proposed to be the main cause of impaired liver function in critically ill patients [1,9,10,11,12]. When liver dysfunction is present, mortality rates of up to 50% have been consistently reported [1,3,9,13]. Hypoxic liver injury is caused by one of the following mechanisms: (1) inadequate oxygen uptake (respiratory dysfunction, “hypoxemic hypoxia”), (2) inadequate oxygen delivery (cardiocirculatory dysfunction, “ischemic hypoxia”), (3) decreased oxygen availability (hemoglobin dysfunction, for example dyshemoglobinemia, “anemic hypoxia”), or (4) increased oxygen consumption (hypermetabolism, for example hyperthermia, “metabolic hypoxia”) [3,9,10,14,15,16]. Regardless of the underlying pathogenesis, the final common pathway is hepatocellular dysfunction due to insufficient cellular oxygen supply to meet the actual metabolic need of mitochondria.

Hypoxic liver injury involves an acute, rapid, excessive (usually 20 times the upper limit of normal), but usually transient and reversible increase in serum transaminase levels in combination with a clinically conclusive setting (e.g., circulatory or respiratory failure) and exclusion of other causes of liver failure [17,18]. 

Hypoxic liver injury (HLI) must be separated from hypoxic cholangiopathy and its late form (secondary) sclerosing cholangiopathy in critically ill patients (SC-CIP) [7,19]. SC-CIP patients usually present with cholestasis manifesting with increased bilirubin and gamma glutamyltransferase (GGT) but only mild or no increases of transaminases [5,19]. SC-CIP appears to be favored by normal anatomy [19], where the biliary tree is due to its embryonic development exclusively supplied by the hepatic artery. Therefore, in contrast to the liver parenchyma with its dual blood supply (hepatic artery and portal vein), the cells of the biliary tract are highly prone to hypoxemia [16,20,21]. 

Since ARDS (acute respiratory distress syndrome) is largely defined by impaired oxygenation leading to hypoxemia [22], it is sufficient to cause SC-CIP by itself [17]. Furthermore, high levels of (total) positive endexpiratory pressure (PEEP; >15 cm H_2_O) and prone positioning, which represent the standard of care in these patients [23,24], have been reported to influence splanchnic perfusion [25,26,27,28], and therefore could also affect oxygen supply on the hepatic artery territory. However, there is also a body of evidence suggesting that liver perfusion is not influenced by PEEP levels [22,29,30]. Regarding extracorporeal membrane oxygenation (ECMO), which is used as a rescue measure in ARDS, there are no data on its influence on splanchnic perfusion available. 

Hypoxemic cell damage most likely occurs when hypoxemia is worst, usually in the onset/early phase of ARDS. In this critical phase, mild impairments of liver function, mild or moderate increases in transaminases, or signs of cholestasis are seemingly of secondary importance or lost in the background noise of the acute phase. Moreover, increases in biomarkers of alleged organ damage are frequent in critical diseases, but their clinical significance is often not distinct [31,32,33]. Interestingly, many biomarkers that can be measured increased as markers for organ damage or supposedly disease-aggravating markers (e.g., IL-6, bile acids) may in fact bring about positive effects [34,35].

We analyzed biomarkers of liver injury and cholestasis in the early phase of ARDS to verify the hypothesis that hypoxic liver injury and hypoxic cholangiopathy are detectable very early in the course of the disease and are associated with the severity of hypoxemia. Furthermore, we hypothesized that higher levels of PEEP, prone position, and ECMO treatment might be associated with the development/course of hypoxic cholangiopathy and/or hypoxic liver injury.

## 2. Materials and Methods

To evaluate the effect of hypoxemic hypoxia on hypoxemic damage to hepatocytes and biliary tree epithelium, we examined a group of patients with ARDS of all severity grades (Berlin-definition) as a sub-study of a multicenter observational trial [36]. Drug induced liver injury was ruled out in all patients; none of the enrolled subjects suffered from underlying or past liver disease based on their thoroughly evaluated past medical history.

The study was approved by the local research ethics board of Georg-August-University Goettingen (IRB No. 18/8/14 on 08.09.2014). Informed consent was obtained from patients, where possible, otherwise from next of kin/legal representatives.

Biomarkers of impaired liver function and cholestasis [37] were extracted from patient records. 

Biomarkers were measured as part of the daily routine, biomarker levels (bilirubin, AST, ALT, GGT) were extracted from patient digital charts on days 0, 3, 5, and 10 after the diagnosis of ARDS, total bile acid levels were analyzed from blood samples taken on the exact same days except for day 10 where no samples were taken. Clinical data were gathered from the department’s patient data management system (PD.M.S; ICCA^®^ Philips Electronics, Hamburg, Germany, Rev. F. 01.01.001), including sex, age, SAPS II, use of prone position (y/n), ECMO treatment (y/n), dialysis (y/n), cause of ARDS (pulmonal/extra-pulmonal), ICU-survival (y/n), time to ICU-death, as well as mean and lowest paO_2_ on days 0, 3, 5, and 10; in terms of respiratory parameters, mean PEEP was extracted on the respective day. Decisions to use prone position or ECMO were at the discretion of the treating team and were not influenced by the study.

Blood samples were analyzed by standard methods on the ARCHITECT cSystem (all assays: Abbott laboratories, Illinois, USA) for Bilirubin (enzyme Diazo reaction), aspartat-aminotransferase (AST) (enzyme NADH oxidation reaction), Alanin-aminotransferase (ALT) (enzyme IFCC method), and gamma glutamyltransferase (GGT) (enzyme L-Gammaglutamyl-S.carboxy-4-nitroanilid substrate). Total bile acid levels were determined by use of a fully validated in-house LC-MS/MS method at the Jena University Hospital (Institute of Clinical Chemistry and Laboratory Medicine) with an Agilent 1200 HPLC-system and a Zorbax Eclipse XDB-C18 column (both: Agilent, Santa Clara, CA, United States).

Statistical analysis was performed using SPSS 26.0 and 27.0 (International Business Machines Corporated [IBM], Armonk, NY, USA). All values were first tested for normal distribution using the Shapiro–Wilk test and outliers were identified. Obtained data were then winsorized to account for outliers (Hemmerich, W. (2019), retrieved from https://statistikguru.de/rechner/winsorizing-rechner.html (accessed on 25 March 2019)). The data was then tested for differences using repeated measures ANOVA with Greenhouse-Geisser or Huynh-Feldt correction as applicable. Significant differences were further tested by Bonferroni adjusted post hoc analysis. Differences between groups were tested for using the Welch test, correlations were tested for by using Kendall-tau test, linear regression was used to test for causation.

## 3. Results

We measured in 70 previously liver-healthy patients; for patient characteristics, see Table 1. Of our studied cohort, 50% of patients were put to prone and 30% were treated with ECMO. The worst Horovitz index was found on day zero and improved significantly with therapy (*p* < 0.001) (Table 2 and Table S1). 

**Figure 1:**
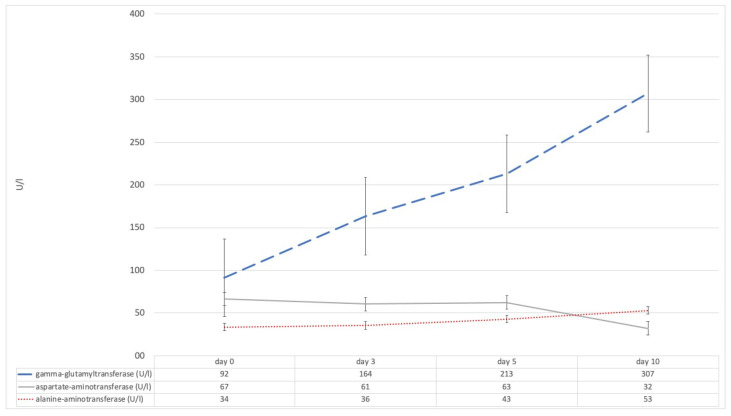
Trends of evaluated biomarkers of hepatitis and cholestasis from the day of diagnosis to day 10 of the course of acute respiratory distress syndrome; statistical significance was only determined for gamma-glutamyltransferase after Bonferroni correction (*p* < 0.001).

**Figure 2:**
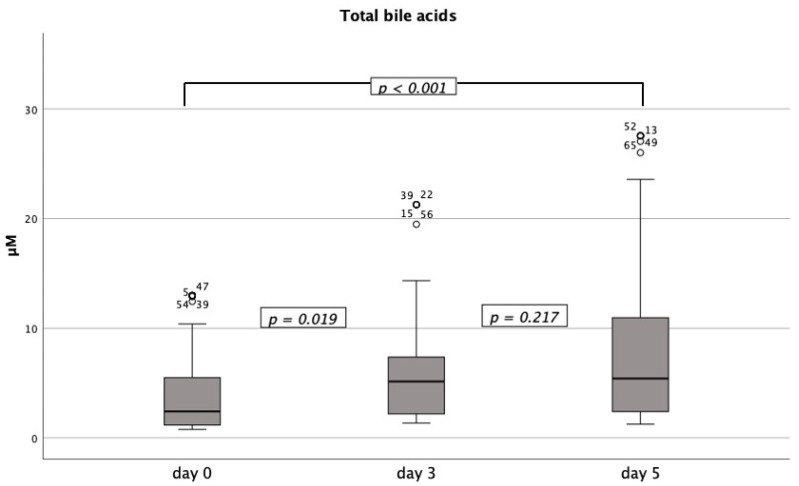
Evolution of total bile acids from day 0 to day 5. Levels of total bile acids were significantly correlated to GGT on day 0 and 3 and in a trend also on day 5.

The total amount of bilirubin was within the normal range for the whole period investigated, this was true for the whole cohort but also after stratification for severity, ECMO, and prone positioning. AST levels increased at a constant level from day zero to day five and then declined to the normal range on day ten; differences in AST levels between days were not significant after Bonferroni correction (Table S1). ALT levels showed the exact opposite kinetic as AST levels; they were within the normal range from day zero to day 5 and then increased to day ten without showing clinically relevant differences (Figure 1). Statistically significant correlations could be found between mean PEEP on the respective days and AST levels on day 3 (*p* = 0.033) as well as ALT levels on day 5 (*p* = 0.015). Furthermore, linear regression found mean PEEP causative for AST levels on day 0 (*p* = 0.038, r^2^ = 0.048) and day 5 (*p* = 0.020, r^2^ = 0.077); no causing effect could be found for prone position or ECMO treatment. GGT levels were highly elevated above the upper limit of reference from day zero and continuously increased throughout the investigated period (Table 2, Figure 1); the increases between days were statistically significant (*p* < 0.001). GGT levels and mean PEEP were only statistically significantly correlated on day 10 with a low effect level (*p* = 0.029); logistic regression revealed no causing effect of mean PEEP, prone position, or ECMO treatment. The total amount of bile acids levels increased significantly from day 0 to day 3 (*p* = 0.019) and day 0 to day 5 (*p* < 0.001), no significant differences were found between day 3 and day 5 (*p* = 0.217) (Figure 2). Total bile acids levels were significantly correlated to GGT on day 0 (*p* < 0.001), day 3 (*p* = 0.02), and in a trend also on day 5 (*p* = 0.055).

There were no clinically significant differences between the groups of patients treated with ECMO and those treated without ECMO, as well as patients proned and those not proned with respect to any of the measured biomarkers (Tables S2 and S3); the cause of ARDS had no influence on the use of ECMO or the application of prone position (Table S4). PaO_2_ on the studied days—mean as well as lowest—were not statistically correlated to the biomarkers investigated.

## 4. Discussion

To our knowledge, this is the first study to exploit a reasonable number of ARDS patients on the day of diagnosis and three more consecutive days during the early phase of the disease with regard to biomarkers of liver damage and cholestasis. Drug induced liver injury can conclusively be ruled out in our cohort. Idiosyncratic hepatotoxicity, which would be the mechanism in most drugs used in intensive/critical care, can be found 5–90 days after application of the drug [37,38]. However, the patients in our cohort were treated in ICU 3.9 days mean prior to ARDS diagnosis/enrollment in our trial (median 3, IQR 1–7), which is too early for idiosyncratic hepatotoxicity to occur. So, in our cohort drug induced liver injury is very unlikely at this stage of the disease and as a cause for the elevations and kinetics of biomarkers we found. We hypothesized that the severity of hypoxemia and consecutive tissue hypoxia characterized by the mean or lowest paO_2_ would provoke or at least foster hypoxic liver injury and hypoxic damage to cholangiocytes. Our results do not confirm this hypothesis, as neither mean paO_2_ nor worst paO_2_ were correlated with bilirubin, transaminases, or GGT. Additionally, the rescue measures that are used during the therapy of ARDS, such as prone positioning and ECMO therapy were not correlated to the measured biomarkers suggesting no influence either way. Only PEEP showed some influence with regard to liver injury and damage to cholangiocytes.

Serum levels of AST and ALT were only moderately elevated and did not reach levels that are considered prove for hypoxic hepatitis (20 times above the upper limit of normal) [17,18], therefore, no hypoxic liver injury was detected in our cohort. This is confirmed by the fact that we found paO_2_ not correlated to transaminases; our data therefore do not support hypoxic hypoxia which would suggest itself in respiratory failure as a reason for liver damage in ARDS. However, the progression of liver damage biomarkers in our study is in good agreement with the progression of hypoxic liver injury recently described [18]. Ebert et al. report an increase in transaminase levels within 12 to 48 h after the causing event—corresponding to day zero in our study—and a decrease of about 50% after resolution of the triggering event (AST day 0: 66.5 U/l, day 10: 32.1 U/l, paO_2_/FiO_2_ ratio increased while mean PEEP decreased during the same period, we consider this prove for improvement). Furthermore, the authors state, that some investigators use lower increases of transaminases to diagnose hypoxic liver injury [18]. Above that, the profile of transaminases we found—AST more increased than ALT—is relatively specific for ischemic/hypoxic hepatitis [39]. 

In contrast to paO_2_, PEEP throughout the investigated days was correlated with AST and ALT levels. Furthermore, linear regression found causality of mean PEEP for AST levels on day 0 and day 5. The underlying mechanism here could be due to splanchnic perfusion impairments due to increased levels of PEEP (ischemic hypoxia) rather than due to gas exchange impairments. Increased PEEP levels could lead to a mechanically conditioned form of decreased splanchnic perfusion [24,40,41]. In this scenario, an increase in PEEP would consecutively increase intraabdominal pressure, leading to a partial functional mechanical obstruction of the liver supplying vessels of the liver; this effect could even be aggravated by prone positioning [41]. Another mechanism with the same result of reduced hepatic perfusion is hepatic congestion due to increased levels of PEEP and consecutive increased intrathoracic pressure. Increases of AST in patients with hepatic congestion have been described before [42], therefore, the increases in AST and the correlation to PEEP can be interpreted in light of this mechanism. However, because ischemic hypoxia from the mechanisms just described is much more relative (partial mechanical obstruction) than hypoxemic hypoxia (absolute reduction of oxygen content), liver damage might be less sustained and consequently biomarkers might be less elevated. Yet, there are no data available that might support different increases of transaminases to define hypoxic liver injury by different mechanisms of hypoxia. However, these findings are in line with our hypothesis that higher levels of PEEP might aggravate hypoxic liver injury. However, the correlation between transaminases and PEEP could also simply be an expression of the severity of the disease.

Gamma glutamyltransferase is an enzyme located on the surface of the biliary epithelium and is essentially released when cells die, therefore, it has been proposed as a sensitive marker of cholangiocyte injury [43]. In our cohort we found high and constantly increasing levels of gamma glutamyltransferase, which in this light could be interpreted as an expression of hypoxic cholangiopathy. However, we did not find correlations between the mean or lowest paO_2_ values and levels of GGT on the respective days, which again makes a hypoxemic hypoxia genesis of the GGT increase seem unlikely. This conclusion is supported by two recent articles that both show the same kinetic of GGT that we also found, but none of their investigated patients explicitly suffered from respiratory failure [5,40]. Kulaksiz et al. describe increases of GGT within one to four weeks after the causing event, however, in their cohort just as in ours, GGT was found increased as early as one day after the initial insult [5].

Mean PEEP in our cohort was correlated to GGT on day 10 though, but only with a low effect level. Above that, linear regression did not find causation of increased GGT levels for PEEP alone, nor for the combination of PEEP and prone position, both factors that have been reported to reduce splanchnic perfusion [24,40,41]. These results largely exclude ischemic hypoxia as a cause of the GGT profile we found in our cohort but suggest that the increase and rise in GGT must be caused by another mechanism. 

Bile acids have been reported to be capable of solubilizing GGT from the membrane surface of cholangiocytes by their detergent action [44,45,46,47]. Since bile acids have been reported to be elevated in serum in many states of disease, including ARDS [48] (Harnisch et al., submitted), this increase in bile acids could be sufficient to solubilize enough GGT from cholangiocyte membranes to increase plasma levels. In fact, we found the serum levels of total bile acids to be correlated to serum levels of GGT on day 0 and 3 and in a trend also on day 5. The reason for this elevation of bile acids in serum in critical illness most likely is due to an adaptive mechanism (Harnisch et al., submitted). If this connection proves to be true, GGT could be viewed as a widely available scout test for regular checks whether the adaptive mechanism of the specific increase in bile acid has already begun. 

If GGT is increased, the total amount of bile acids should be measured to exclude other reasons than the mechanism proposed here. If total bile acids are also found to be increased, we highly recommend quantifying individual bile acids and their conjugation products, because their individual composition could help to differentiate an adaptive from a maladaptive response to critical illness (Harnisch et al., submitted), and above that could also help predict outcome (Harnisch et al., manuscript in preparation). Obviously further extensive research is necessary to verify this hypothesis. 

Limitations: We report a single center observational trial on a reasonable but still limited number of patients, which makes our findings prone to bias and commands caution in interpreting the results. We did not break the GGT into subspecies, which could underline our hypothesis. Moreover, we did not evaluate for the period of hypoxemia but only the day we took the serum specimen. This might have veiled another layer in addition to the severity of hypoxemia, i.e., the duration of hypoxemia. Further trials, ideally in a multicenter setting, on a larger number of patients, including a broader range of biomarkers and dynamic liver tests of liver function are needed to confirm our findings.

## 5. Conclusions

In our cohort of 70 patients with ARDS of all severity levels, we found no evidence that hypoxic liver injury or hypoxic damage to cholangiocytes was caused by the severity of hypoxemia in ARDS patients during the very early phase of the disease. Additionally, the mean PEEP level, prone positioning, and ECMO treatment did not have an impact in this regard. Increases in GGT can be interpreted as an indirect marker of increased plasma levels of bile acid, probably in the sense of an adaptive response mechanism to this critical condition. Therefore, GGT could be viewed as a scout that, if elevated, should blaze the trail for further testing of total and finally individual bile acid levels to possibly differentiate an adaptive response from a maladaptive one. Further studies will have to evaluate if our results can be reproduced.

## Figures and Tables

**Table 1:** Subject characteristics.

Parameter	Mean ± SD		
Age (years)	58.0 ± 14.9		
Female:male	27:43		
SAPS II	40.0 ± 12.1		
ARDS severity	mild	moderate	severe
	4	38	28
Deceased in ICU	19		
Cause of ARDS	pulmonal	extrapulmonal	
	40	30	
ECMO therapy	21 (30%)		

**Table 2:** Median and interquartile range for measured biomarkers as well as paO^2^ levels and mean PEEP of all patients after winsorization.

Parameter	Day 0	Day 3	Day 5	Day 10
Total bilirubin (mg/dl)	0.6 (0.4–1.275)	0.6 (0.4–1.1)	0.7 (0.4–1.5)	0.9 (0.5–1.875)
Gamma-glutamyltransferase (U/l)	91.5 (47–202.5)	163.5 (86.75–253.25)	213 (124–355.5)	307 (143–767)
Aspartat aminotransferase (U/l)	66.5 (32.5–119)	60.5 (38–119.5)	63.5 (32.25–106.5)	32.1 (32.05–32.1)
Alanin aminotransferase (U/l)	33.5 (17.75–83.25)	35.5 (20.75–69.25)	43 (22–75)	53 (28.5–90)
Total bile acids (µM/l)	2.42 (1.16–5.69)	5.14 (2.7–7.42)	5.41 (2.40–10.99)	n.a.
lowest paO_2_ (mmHg)	60 (53–71.5)	65.5 (58.25–73)	65 (57.25–74.75)	68 (60–79)
mean paO_2_ (mmHg)	84.25 (77.1–95.38)	84.35 (76.7–93.33)	81.05 (74.53–91.13)	86.15 (78.03–97.53)
mean PEEP (cmH_2_O)	11.65 (9.78–13.83)	10.7 (8–12.9)	10.25 (8–12.93)	10 (8.3–12)

## Data Availability

Data can be obtained from the corresponding author upon reasonable request.

## Supplementary Materials

The following are available online at https://www.mdpi.com/article/10.3390/diagnostics11122356/s1, Table S1: Results of repeated measures ANOVA, including post hoc test for all patients, Table S2: Welch-Test ECMO vs. non-ECMO treatment, Table S3: Welch test of subjects placed prone vs. subjects kept supine, Table S4: Correlations (Kendall-tau test) between measured biomarkers, general characteristics, paO^2^, and PEEP.

## References

[1] Fuhrmann V., Kneidinger N., Herkner H., Heinz G., Nikfardjam M., Bojic A., Schellongowski P., Angermayr B., Schoniger-Hekele M., Madl C. (2011). Impact of hypoxic hepatitis on mortality in the intensive care unit. Intensive Care Med..

[2] Kramer L., Jordan B., Druml W., Bauer P., Metnitz P.G., Austrian Epidemiologic Study on Intensive Care A.S.G. (2007). Incidence and prognosis of early hepatic dysfunction in critically ill patients—A prospective multicenter study. Crit. Care Med..

[3] Birrer R., Takuda Y., Takara T. (2007). Hypoxic hepatopathy: Pathophysiology and prognosis. Intern. Med..

[4] Kortgen A., Paxian M., Werth M., Recknagel P., Rauchfuss F., Lupp A., Krenn C.G., Muller D., Claus R.A., Reinhart K. (2009). Prospective assessment of hepatic function and mechanisms of dysfunction in the critically ill. Shock.

[5] Kulaksiz H., Heuberger D., Engler S., Stiehl A. (2008). Poor outcome in progressive sclerosing cholangitis after septic shock. Endoscopy.

[6] Lone N.I., Walsh T.S. (2012). Impact of intensive care unit organ failures on mortality during the five years after a critical illness. Am. J. Respir. Crit. Care Med..

[7] Kirchner G.I., Scherer M.N., Obed A., Ruemmele P., Wiest R., Froh M., Loss M., Schlitt H.J., Scholmerich J., Gelbmann C.M. (2011). Outcome of patients with ischemic-like cholangiopathy with secondary sclerosing cholangitis after liver transplantation. Scand. J. Gastroenterol..

[8] Taramasso L., Vena A., Bovis F., Portunato F., Mora S., Dentone C., Delfino E., Mikulska M., Giacobbe D.R., De Maria A. (2020). Higher Mortality and Intensive Care Unit Admissions in COVID-19 Patients with Liver Enzyme Elevations. Microorganisms.

[9] Henrion J., Schapira M., Luwaert R., Colin L., Delannoy A., Heller F.R. (2003). Hypoxic hepatitis: Clinical and hemodynamic study in 142 consecutive cases. Medicine.

[10] Seeto R.K., Fenn B., Rockey D.C. (2000). Ischemic hepatitis: Clinical presentation and pathogenesis. Am. J. Med..

[11] Whitehead M.W., Hawkes N.D., Hainsworth I., Kingham J.G. (1999). A prospective study of the causes of notably raised aspartate aminotransferase of liver origin. Gut.

[12] Johnson R.D., O'Connor M.L., Kerr R.M. (1995). Extreme serum elevations of aspartate aminotransferase. Am. J. Gastroenterol..

[13] Fuhrmann V., Kneidinger N., Herkner H., Heinz G., Nikfardjam M., Bojic A., Schellongowski P., Angermayr B., Kitzberger R., Warszawska J. (2009). Hypoxic hepatitis: Underlying conditions and risk factors for mortality in critically ill patients. Intensive Care Med..

[14] Henrion J., Minette P., Colin L., Schapira M., Delannoy A., Heller F.R. (1999). Hypoxic hepatitis caused by acute exacerbation of chronic respiratory failure: A case-controlled, hemodynamic study of 17 consecutive cases. Hepatology.

[15] Henrion J., Colin L., Schapira M., Heller F.R. (1997). Hypoxic hepatitis caused by severe hypoxemia from obstructive sleep apnea. J. Clin. Gastroenterol..

[16] Leonhardt S., Veltzke-Schlieker W., Adler A., Schott E., Hetzer R., Schaffartzik W., Tryba M., Neuhaus P., Seehofer D. (2015). Trigger mechanisms of secondary sclerosing cholangitis in critically ill patients. Crit. Care.

[17] Fuhrmann V., Jager B., Zubkova A., Drolz A. (2010). Hypoxic hepatitis-epidemiology, pathophysiology and clinical management. Wien. Klin. Wochenschr..

[18] Ebert E.C. (2006). Hypoxic liver injury. Mayo Clin. Proc..

[19] Gelbmann C.M., Rummele P., Wimmer M., Hofstadter F., Gohlmann B., Endlicher E., Kullmann F., Langgartner J., Scholmerich J. (2007). Ischemic-like cholangiopathy with secondary sclerosing cholangitis in critically ill patients. Am. J. Gastroenterol..

[20] Li M.K., Crawford J.M. (2004). The pathology of cholestasis. Semin Liver Dis.

[21] Kobayashi S., Nakanuma Y., Matsui O. (1994). Intrahepatic Peribiliary Vascular Plexus in Various Hepatobiliary Diseases—A Histological Survey. Hum. Pathol..

[22] Saner F.H., Olde Damink S.W.M., Pavlaković G., Sotiropoulos G.C., Radtke A., Treckmann J., Beckebaum S., Cicinnati V., Paul A. (2010). How far can we go with positive end-expiratory pressure (PEEP) in liver transplant patients?. J. Clin. Anesth..

[23] Bein T., Grasso S., Moerer O., Quintel M., Guerin C., Deja M., Brondani A., Mehta S. (2016). The standard of care of patients with ARDS: Ventilatory settings and rescue therapies for refractory hypoxemia. Intensive Care Med..

[24] Gattinoni L., Quintel M. (2016). How ARDS should be treated. Crit. Care.

[25] Putensen C., Wrigge H., Hering R. (2006). The effects of mechanical ventilation on the gut and abdomen. Curr. Opin. Crit. Care.

[26] Michelet P., Roch A., Gainnier M., Sainty J.M., Auffray J.P., Papazian L. (2005). Influence of support on intra-abdominal pressure, hepatic kinetics of indocyanine green and extravascular lung water during prone positioning in patients with ARDS: A randomized crossover study. Crit. Care.

[27] Bonnert F., Richard C., Glaser P., Lafay M. (1982). Changes in hepatic flow induced by continuous positive pressure ventilation in critically ill patients. Crit. Care Med..

[28] Chikhani M., Evans D.L., Blatcher A.W., Jackson A.P., Guha I.N., Aithal G.P., Moppett I.K. (2016). The effect of prone positioning with surgical bolsters on liver blood flow in healthy volunteers. Anaesthesia.

[29] Saner F.H., Pavlaković G., Gu Y., Fruhauf N.R., Paul A., Radtke A., Nadalin S., Malagó M., Broelsch C.E. (2006). Does PEEP impair the hepatic outflow in patients following liver transplantation?. Intensive Care Med..

[30] Brienza N., Revelly J.P., Ayuse T., Robotham J.L. (1995). Effects of PEEP on liver arterial and venous blood flows. Am. J. Respir. Crit. Care Med..

[31] Kimberly W.T. (2012). Biomarkers in neurocritical care. Neurotherapeutics.

[32] Ackland G.L., Mythen M.G. (2007). Novel biomarkers in critical care: Utility or futility?. Crit. Care.

[33] Kibe S., Adams K., Barlow G. (2011). Diagnostic and prognostic biomarkers of sepsis in critical care. J. Antimicrob. Chemother..

[34] Leonhardt J., Haider R.S., Sponholz C., Leonhardt S., Drube J., Spengler K., Mihaylov D., Neugebauer S., Kiehntopf M., Lambert N.A. (2021). Circulating Bile Acids in Liver Failure Activate TGR5 and Induce Monocyte Dysfunction. Cell. Mol. Gastroenterol. Hepatol..

[35] Rose-John S. (2012). IL-6 trans-signaling via the soluble IL-6 receptor: Importance for the pro-inflammatory activities of IL-6. Int. J. Biol. Sci..

[36] Brandstetter S., Dodoo-Schittko F., Blecha S., Sebok P., Thomann-Hackner K., Quintel M., Weber-Carstens S., Bein T., Apfelbacher C. (2015). Influence of quality of care and individual patient characteristics on quality of life and return to work in survivors of the acute respiratory distress syndrome: Protocol for a prospective, observational, multi-centre patient cohort study (DACAPO). BMC Health Serv. Res..

[37] Kumachev A., Wu P.E. (2021). Drug-induced liver injury. CMAJ.

[38] Hoofnagle J.H., Bjornsson E.S. (2019). Drug-Induced Liver Injury—Types and Phenotypes. N. Engl. J. Med..

[39] Woreta T.A., Alqahtani S.A. (2014). Evaluation of abnormal liver tests. Med. Clin. N. Am..

[40] Lin T., Qu K., Xu X., Tian M., Gao J., Zhang C., Di Y., Zhang Y., Liu C. (2014). Sclerosing cholangitis in critically ill patients: An important and easily ignored problem based on a German experience. Front. Med..

[41] Geiger K., Georgieff M., Lutz H. (1986). Side effects of positive pressure ventilation on hepatic function and splanchnic circulation. Int.J. Clin. Monit. Comput..

[42] Killip T., Payne M.A. (1960). High serum transaminase activity in heart disease. Circulatory failure and hepatic necrosis. Circulation.

[43] Visentin M., Lenggenhager D., Gai Z., Kullak-Ublick G.A. (2018). Drug-induced bile duct injury. Biochim. Biophys. Acta Mol. Basis Dis..

[44] Hirata E., Inoue M., Morino Y. (1984). Mechanism of biliary secretion of membranous enzymes: Bile acids are important factors for biliary occurrence of gamma-glutamyltransferase and other hydrolases. J. Biochem..

[45] Arrese M., Pizarro M., Solís N., Koenig C., Accatino L. (1995). Enhanced biliary excretion of canalicular membrane enzymes in ethynylestradiol-inducedcholestasis. Biochem. Pharmacol..

[46] Accatino L., Figueroa C., Pizarro M., Solís N. (1995). Enhanced biliary excretion of canalicular membrane enzymes in estrogen-induced and obstructive cholestasis, and effects of different bile acids in the isolated perfused rat liver. J. Hepatol..

[47] Schlaeger R., Haux P., Kattermann R. (1982). Studies on the mechanism of the increase in serum alkaline phosphatase activity in cholestasis: Significance of the hepatic bile acid concentration for the leakage of alkaline phosphatase from rat liver. Enzyme.

[48] Harnisch L.O., Moerer O. (2020). The Specific Bile Acid Profile of Shock: A Hypothesis Generating Appraisal of the Literature. J. Clin. Med..

