# Supplementary material for: Biomarkers of Cholestasis and Liver Injury in the Early Phase of Acute Respiratory Distress Syndrome and Their Pathophysiological Value
Source: Diagnostics (Basel). 2021 Dec 14;11(12):2356. doi: 10.3390/diagnostics11122356 (PMC8699895; doi:10.3390/diagnostics11122356)
Supplement: Supplementary file 1 [file diagnostics-11-02356-s001.zip › diagnostics-1425230-supplementary.pdf]

Table S1: Results of repeated measures ANOVA including post-hoc test for all patients

Parameter	Repeated measure ANOVA	Post-hoc test (Bonferroni)
**Total bilirubin**	F(1.03, 101.23) = 2.41, p = 0.371	
Days 0-3		p = 1.0
Days 3-5		p = 0.58
Days 0-5		p = 1.0
Days 3-10		p = 1.0
Days 5-10		p = 1.0
Days 0-10		p = 1.0
**Gamma glutamyltransferase**	*F(25.61, 59.16) = 1.44, p < 0.001**	
Days 0-3		***p = 0.003****
Days 3-5		***p < 0.001****
Days 0-5		***p < 0.001****
Days 3-10		***p < 0.001****
Days 5-10		***p = 0.001****
Days 0-10		***p < 0.001****
**Aspartat aminotransferase**	*F(4.4, 24.0) = 3.0, p = 0.013**	
Days 0-3		p = 1.0
Days 3-5		p = 1.0
Days 0-5		p = 1.0
Days 3-10		p = 0.445
Days 5-10		p = 0.125
Days 0-10		p = 0.180
**Alanin aminotransferase**	*F(3.12, 80.67) = 2.07, p = 0.048**	
Days 0-3		***p = 0.014****
Days 3-5		p = 1.0
Days 0-5		p = 0.229
Days 3-10		p = 1.0
Days 5-10		p = 1.0
Days 0-10		p = 1.0
**mean paO^2**	F(1.815, 95.208) = 1.73, p = 0.173	
Days 0-3		p = 0.881
Days 3-5		p = 1.0
Days 0-5		p = 0.543
Days 3-10		p = 1.0
Days 5-10		p = 0.364
Days 0-10		p = 1.0

**lowest paO^2**	*F(4.051, 165) = 3, p = 0.008**	
Days 0-3		p = 0.112
Days 3-5		p = 1.0
Days 0-5		p = 0.208
Days 3-10		p = 1.0
Days 5-10		p = 1.0
Days 0-10		***p = 0.036****
**mean PEEP (cmH_2O)**	*F(4.811, 87.46) = 1.9, p = 0.012**	
Days 0-3		p = 0.238
Days 3-5		p = 0.731
Days 0-5		p = 0.108
Days 3-10		p = 0.410
Days 5-10		p = 1.0
Days 0-10		p = 0.055

Table S2: Welch-Test ECMO vs. Non-ECMO treatment, data are presented as mean and standard deviation, median and interquartile range respectively as applicable

<table>
<tr><th>Parameter</th><th colspan="6">ECMO treatment</th><th colspan="6">Non-ECMO treatment</th><th>p-value</th></tr>
<tr><td>Number of patients</td><td colspan="6">21</td><td colspan="6">49</td><td>-</td></tr>
<tr><td>Age (years)</td><td colspan="6">53.57 ± 13.80</td><td colspan="6">59.58 ± 15.43</td><td>0.117</td></tr>
<tr><td>SAPS II</td><td colspan="6">40.81 ± 12.08</td><td colspan="6">40.13 ± 12.40</td><td>0.785</td></tr>
<tr><td>Survival (count)</td><td colspan="6">10</td><td colspan="6">41</td><td>0.007*</td></tr>
<tr><td>ARDS severity (Berlin-definition)</td><td colspan="2">0</td><td colspan="2">9</td><td colspan="2">12</td><td colspan="2">4</td><td colspan="2">29</td><td colspan="2">16</td><td>0.024*</td></tr>
<tr><td>Cause of ARDS (pulmonary/extra-pulmonary)</td><td colspan="3">14</td><td colspan="3">7</td><td colspan="3">26</td><td colspan="3">23</td><td>0.293</td></tr>
<tr><td>Days in ICU (days)</td><td colspan="6">21 (14- 34.5)</td><td colspan="6">24.5 (16- 37.5)</td><td>0.958</td></tr>
<tr><td>Days on ventilator (days)</td><td colspan="6">20 (12- 34.5)</td><td colspan="6">21.5 (13.5- 27.75)</td><td>0.783</td></tr>
<tr><td>Days free of invasive ventilation (days)</td><td colspan="6">0 (0- 0)</td><td colspan="6">2 (0- 4)</td><td>0.802</td></tr>
<tr><td>mean paO^2 day 0</td><td colspan="6">89.65 (71.13- 121.10)</td><td colspan="6">82.9 (77.30- 90.43)</td><td>0.131</td></tr>
<tr><td>mean paO^2 day 3</td><td colspan="6">81.85 (72.30- 95.58)</td><td colspan="6">88.1 (78.2- 93.18)</td><td>0.440</td></tr>
<tr><td>mean paO^2 day 5</td><td colspan="6">80 (72.53- 84.45)</td><td colspan="6">84.4 (75.35- 91.7)</td><td>0.874</td></tr>
<tr><td>mean paO^2 day 10</td><td colspan="6">83.25 (76- 92.48)</td><td colspan="6">87.6 (78.9- 98.05)</td><td>0.140</td></tr>
</table>

**lowest paO^2 day 0**	53 (47.75- 71)	61.5 (54- 72)	0.791
**lowest paO^2 day 3**	58.50 (55 – 67.5)	70.5 (652.77.25)	0.187
**lowest paO^2 day 5**	63.5 (57.5- 72.5)	65.5 (57- 75)	0.514
**lowest paO^2 day 10**	67 (57.5- 77.5)	70.5 (59.75- 89.2)	0.524
**mean PEEP day 0 (cmH_2O)**	12 (9.8- 13.95)	11.5 (9.75- 13.65)	0.772
**mean PEEP day 3 (cmH_2O)**	12 (9.4- 13.7)	10 (6.7- 12.25)	**0.041***
**mean PEEP day 5 (cmH_2O)**	11.7 (9- 14.25)	10 (7.45- 12.3)	0.125
**mean PEEP day 10 (cmH_2O)**	10.9 (8.85- 12)	9.75 (7.95- 11.7)	0.535
**Total bilirubin day 0 (mg/dl)**	0.6 (0.45- 0.95)	0.6 (0.38- 1.7)	0.223
**Total bilirubin day 3 (mg/dl)**	0.6 (0.45- 1.15)	0.5 (0.38- 1.1)	0.843
**Total bilirubin day 5 (mg/dl)**	0.8 (0.45- 2.1)	0.55 (0.3- 1)	0.273
**Total bilirubin day 10 (mg/dl)**	0.9 (0.5- 2.68)	0.9 (0.5- 1.5)	0.598
**Gamma glutamyltransferase day 0 (U/l)**	56 (44.5- 197.5)	94.5 (58.5- 202.5)	0.815
**Gamma glutamyltransferase day 3 (U/l)**	172 (62.5- 322.5)	154 (89.35- 265)	0.757
**Gamma glutamyltransferase day 5 (U/l)**	257 (126- 446)	202.5 (97.5- 317.25)	0.161
**Gamma glutamyltransferase day 10 (U/l)**	493 (143- 767)	272 (128.25- 786.75)	0.451
**Aspartat aminotransferase day 0 (U/l)**	75 (51.5- 75)	48 (30- 116)	0.374
**Aspartat aminotransferase day 3 (U/l)**	83.5 (47.25- 152)	57 (37- 107)	0.098
**Aspartat aminotransferase day 5 (U/l)**	70.5 (50.75- 119.75)	52.5 (32.5- 95)	0.198
**Aspartat aminotransferase day 10 (U/l)**	32.05 (32- 32.05)	32.1 (32.1- 32.1)	0.372
**Alanin aminotransferase day 0 (U/l)**	31 (17.5- 96.5)	17.5 (34- 64.5)	0.748
**Alanin aminotransferase day 3 (U/l)**	36 (23.5- 54.5)	35 (19- 77.5)	0.966
**Alanin aminotransferase day 5 (U/l)**	49 (28.5- 79)	40 (16.25- 72.5)	0.491
**Alanin aminotransferase day 10 (U/l)**	63 (30-95)	52.5 (20.75- 90.7)	0.528

Table S3: Welch-Test of subjects put in prone vs. subjects kept supine, data are presented as mean and standard deviation, median and interquartile range respectively as applicable

Parameter	Prone position	No prone position	p-value
**Number of patients**	35	35	-

<table>
<tr><td>Age (years)</td><td colspan="6">56 (43- 67)</td><td colspan="6">63 (53- 74)</td><td>0.148</td></tr>
<tr><td>SAPS II</td><td colspan="6">39.17 ± 13.90</td><td colspan="6">41.53 ± 10.27</td><td>0.484</td></tr>
<tr><td>Survival (count)</td><td colspan="6">22</td><td colspan="6">29</td><td>0.062</td></tr>
<tr><td>ARDS severity (Berlin-definition)</td><td colspan="2">0</td><td colspan="2">18</td><td colspan="2">17</td><td colspan="2">4</td><td colspan="2">20</td><td colspan="2">11</td><td>0.041*</td></tr>
<tr><td>Cause of ARDS (pulmonary/extra-pulmonary)</td><td colspan="3">20</td><td colspan="3">15</td><td colspan="3">20</td><td colspan="3">15</td><td>1.0</td></tr>
<tr><td>Days on ICU (days)</td><td colspan="6">26 (17- 38)</td><td colspan="6">21.5 (14- 34.5)</td><td>0.138</td></tr>
<tr><td>Days in ventilator (days)</td><td colspan="6">22 (15- 34)</td><td colspan="6">21 (10.5- 27.25)</td><td>0.273</td></tr>
<tr><td>Days free of invasive ventilation (days)</td><td colspan="6">0 (0- 3)</td><td colspan="6">1 (0- 2.5)</td><td>0.722</td></tr>
<tr><td>mean paO^2 day 0</td><td colspan="6">86.95 (80.58- 111.30)</td><td colspan="6">82.25 (72- 91.03)</td><td>0.390</td></tr>
<tr><td>mean paO^2 day 3</td><td colspan="6">88.25 (79.63- 93.83)</td><td colspan="6">79.15 (72.98- 91.8)</td><td>0.360</td></tr>
<tr><td>mean paO^2 day 5</td><td colspan="6">79.7 (72.88- 85.5)</td><td colspan="6">87 (76.53- 91.8)</td><td>0.319</td></tr>
<tr><td>mean paO^2 day 10</td><td colspan="6">85.3 (78.08- 92.53)</td><td colspan="6">91.25 (75.9- 98.58)</td><td>0.662</td></tr>
<tr><td>lowest paO^2 day 0</td><td colspan="6">60 (52.75- 70)</td><td colspan="6">60 (51.75- 72.25)</td><td>0.431</td></tr>
<tr><td>lowest paO^2 day 3</td><td colspan="6">66 (56.75- 77)</td><td colspan="6">63.5 (59.75- 73)</td><td>0.775</td></tr>
<tr><td>lowest paO^2 day 5</td><td colspan="6">62.5 (55.75- 71.5)</td><td colspan="6">70.5 (57.75- 77)</td><td>0.027*</td></tr>
<tr><td>lowest paO^2 day 10</td><td colspan="6">68.5 (61.75- 74.75)</td><td colspan="6">67.5 (57.75- 82.75)</td><td>0.938</td></tr>
<tr><td>mean PEEP day 0 (cmH$_2$O)</td><td colspan="6">12.4 (10.7- 15)</td><td colspan="6">10.4 (8.5- 12)</td><td>0.014*</td></tr>
<tr><td>mean PEEP day 3 (cmH$_2$O)</td><td colspan="6">12 (8.8- 13.7)</td><td colspan="6">10 (6.9- 12.03)</td><td>0.056</td></tr>
<tr><td>mean PEEP day 5 (cmH$_2$O)</td><td colspan="6">10.3 (8.3- 12.9)</td><td colspan="6">10 (7.8- 13)</td><td>0.511</td></tr>
<tr><td>mean PEEP day 10 (cmH$_2$O)</td><td colspan="6">10.9 (8.7- 12)</td><td colspan="6">9.75 (8- 11.45)</td><td>0.593</td></tr>
<tr><td>Total bilirubin day 0 (mg/dl)</td><td colspan="6">0.65 (0.4- 1.2)</td><td colspan="6">0.6 (0.4- 1.35)</td><td>0.494</td></tr>
<tr><td>Total bilirubin day 3 (mg/dl)</td><td colspan="6">0.6 (0.4- 1.13)</td><td colspan="6">0.5 (0.4- 0.95)</td><td>0.894</td></tr>
<tr><td>Total bilirubin day 5 (mg/dl)</td><td colspan="6">0.75 (0.4- 1.58)</td><td colspan="6">0.5 (0.3- 1.1)</td><td>0.904</td></tr>
<tr><td>Total bilirubin day 10 (mg/dl)</td><td colspan="6">1.0 (0.5- 2.3)</td><td colspan="6">0.9 (0.6- 1.25)</td><td>0.593</td></tr>
<tr><td>Gamma glutamyltransferase day 0 (U/l)</td><td colspan="6">80 (47- 196.5)</td><td colspan="6">94.5 (43.75- 208.5)</td><td>0.692</td></tr>
<tr><td>Gamma glutamyltransferase day 3 (U/l)</td><td colspan="6">150 (76.5- 228)</td><td colspan="6">180 (101.5- 286)</td><td>0.280</td></tr>
<tr><td>Gamma glutamyltransferase day 5 (U/l)</td><td colspan="6">213 (128.5- 356)</td><td colspan="6">206.5 (96.25- 312)</td><td>0.493</td></tr>
<tr><td>Gamma glutamyltransferase day 10 (U/l)</td><td colspan="6">435 (148.75- 864.5)</td><td colspan="6">256 (70- 687)</td><td>0.291</td></tr>
</table>

**Aspartat aminotransferase day 0 (U/l)**	83 (42- 166)	50 (30-84)	**0.027***
**Aspartat aminotransferase day 3 (U/l)**	68.5 (40.8- 138.8)	57 (36- 91.25)	0.162
**Aspartat aminotransferase day 5 (U/l)**	62.5 (40- 110)	61 (31- 99)	0.665
**Aspartat aminotransferase day 10 (U/l)**	32.1 (32.05- 32.1)	32.1 (24.53- 32.1)	0.394
**Alanin aminotransferase day 0 (U/l)**	34 (17 -95)	33 (18- 83)	0.765
**Alanin aminotransferase day 3 (U/l)**	38 (21- 65)	25 (19- 76)	0.986
**Alanin aminotransferase day 5 (U/l)**	43 (22.5- 71)	43 (16.75- 85.5)	0.779
**Alanin aminotransferase day 10 (U/l)**	61 (45.75- 105)	39 (15.5- 84.5)	0.096

Table S4: Correlations (Kendall-tau test) between measured biomarkers, general characteristics, paO^2, and PEEP. Significant correlations are marked "" for $p < 0.05$, "**" for $p < 0.001$.*

		**Severity Berlin definition**	**Prone position**	**Pulmonal cause**	**ECMO therapy**	**Survival**	**mean paO^2 day 0**	**mean paO^2 day 3**	**mean paO^2 day 5**	**mean paO^2 day 10**	**lowest paO^2 day 0**	**lowest paO^2 day 3**	**lowest paO^2 day 5**	**lowest paO^2 day 10**	**Mean PEEP day 0**	**Mean PEEP day 3**	**Mean PEEP day5**	**Mean PEEP day 10**
**Total bilirubin day 0**	**r-value**	0,119	-0,006	-0,050	0,025	-0,058	-0,222	-0.069	-0.032	0.010	-0.108	0.078	0.105	-0.122	-0,051	-0,059	-0,105	0,047
	**p-value**	0,238	0,953	0,628	0,811	0,576	0,065	0.571	0.797	0.941	0.375	0.523	0.399	0.371	0,556	0,500	0,240	0,657
**Total bilirubin day 3**	**r-value**	0,141	0,000	-0,097	0,066	-0,094	-0.266	-0.064	-0.031	0.119	-0.113	0.113	0.071	0.0056	-0,083	-0,089	-0,156	-0,049
	**p-value**	0,162	1,000	0,351	0,522	0,365	0.026*	0.599	0.807	0.383	0.353	0.352	0.573	0.970	0,338	0,312	0,081	0,638
**Total bilirubin day 5**	**r-value**	0,129	0,096	-0,178	0,145	-0,077	-0.226	0.085	-0.085	0.068	-0.122	0.157	0.065	0.009	0,031	-0,009	-0,054	0,022
	**p-value**	0,214	0,367	0,094	0,170	0,465	0.071	0.503	0.506	0.622	0.331	0.211	0.614	0.950	0,728	0,918	0,550	0,834
**Total bilirubin day 10**	**r-value**	-0,143	0,069	-0,246	0,044	-0,084	-0.105	0.208	-0.124	0.008	-0.066	0.145	0.015	0.031	0,028	0,072	0,103	0,290*
	**p-value**	0,262	0,596	0,057	0,735	0,516	0.499	0.175	0.428	0.960	0.669	0.349	0.923	0.844	0,799	0,508	0,343	0,013
**AST day 0**	**r-value**	0,249*	0,192	0,026	0,148	0,044	-0.017	0.004	-0.207	-0.036	-0.148	-0.048	-0.125	-0.044	0,152	0,162	0,027	-0,047
	**p-value**	0,011	0,055	0,798	0,138	0,658	0.892	0.974	0.096	0.793	0.222	0.692	0.316	0.745	0,069	0,056	0,756	0,645
**AST day 3**	**r-value**	0,188	0,135	0,107	0,193	0,076	-0.204	0.029	-0.065	-0.030	-0.129	0.028	0.046	-0.066	0,142	0,188*	0,146	0,023
	**p-value**	0,069	0,196	0,305	0,065	0,469	0.106	0.819	0.618	0.835	0.309	0.828	0.721	0.642	0,106	0,033	0,103	0,834
**AST day 5**	**r-value**	0,197	0,059	0,110	0,157	0,023	-0.266	-0.008	-0.417	0.010	-0.230	0.026	-0.191	-0.110	0,174	0,216*	0,259**	0,191
	**p-value**	0,064	0,584	0,310	0,146	0,834	0.040*	0.953	0.001*	0.946	0.077	0.845	0.151	0.449	0,056	0,018	0,005	0,075
**AST day 10**	**r-value**	0,115	0,115	-0,577	-0,276	0,000	-0.302	0.183	-0.137	0.297	-0.275	0.046	-0.115	-0.068	-0,236	-0,306	-0,131	0,258
	**p-value**	0,729	0,737	0,094	0,423	1,000	0.402	0.638	0.725	0.438	0.474	0.907	0.769	0.861	0,449	0,309	0,663	0,499
**ALT day 0**	**r-value**	0,201*	0,022	0,029	0,027	0,060	0.045	0.039	-0.238	0.041	-0.184	0.026	-0.091	0.067	0,072	0,147	0,066	-0,028

	p-value	0,040	0,828	0,771	0,788	0,552	0.709	0.750	0.054	0.763	0.128	0.829	0.468	0.625	0,390	0,082	0,450	0,782
ALT day 3	r-value	0,073	0,014	-0,005	0,033	0,177	0.003	0.043	-0.069	-0.095	-0.092	0.147	0.044	-0.064	0,090	0,126	0,121	0,081
	p-value	0,456	0,888	0,962	0,743	0,077	0.983	0.725	0.538	0.485	0.451	0.224	0.728	0.637	0,285	0,138	0,163	0,434
ALT day 5	r-value	0,120	-0,014	0,013	0,105	0,153	-0.053	0.041	-0.219	0.043	-0.234	0.023	-0.027	0.007	0,144	0,199*	0,216*	0,142
	p-value	0,240	0,895	0,900	0,312	0,143	0.677	0.745	0.085	0.754	0.061	0.857	0.836	0.959	0,098	0,023	0,015	0,172
ALT day 10	r-value	0,111	0,236	-0,065	0,061	0,268*	0.096	0.233	-0.275	0.033	-0.062	0.085	-0.186	0.141	0,218	0,229*	0,259*	0,211
	p-value	0,399	0,075	0,623	0,645	0,043	0.550	0.143	0.086	0.838	0.698	0.596	0.251	0.384	0,052	0,041	0,020	0,081
GGT day 0	r-value	0,047	-0,039	0,045	0,023	-0,059	-0.158	-0.076	-0.156	0.038	-0.057	-0.148	-0.135	0.023	-0,035	-0,034	-0,043	0,022
	p-value	0,627	0,694	0,652	0,817	0,556	0.193	0.532	0.211	0.779	0.637	0.220	0.281	0.869	0,673	0,689	0,617	0,832
GGT day 3	r-value	0,002	-0,107	-0,036	0,010	0,081	-0.184	-0.088	-0.069	0.065	-0.046	-0.031	0.001	0.121	0,005	-0,009	0,024	0,160
	p-value	0,986	0,282	0,717	0,918	0,416	0.128	0.467	0.580	0.634	0.706	0.802	0.994	0.374	0,955	0,917	0,781	0,120
GGT day 5	r-value	0,071	0,052	0,067	0,126	0,033	-0.100	0.115	-0.113	0.172	-0.098	0.060	-0.021	0.215	0,158	0,128	0,074	0,177
	p-value	0,486	0,613	0,516	0,225	0,751	0.430	0.360	0.378	0.208	0.043	0.635	0.868	0.115	0,068	0,141	0,405	0,086
GGT day 10	r-value	0,020	0,155	-0,069	0,115	0,211	0.043	0.164	-0.018	0.092	0.125	0.017	-0.042	0.159	0,202	0,279*	0,296**	0,259*
	p-value	0,875	0,230	0,590	0,371	0,102	0.787	0.294	0.908	0.557	0.424	0.911	0.790	0.307	0,063	0,010	0,006	0,029
